# Genome Sequences of Two Lytic Staphylococcus aureus Bacteriophages Isolated from Wastewater

**DOI:** 10.1128/mra.00954-22

**Published:** 2022-11-21

**Authors:** Joshua J. Iszatt, Alexander N. Larcombe, Luke W. Garratt, Stephen M. Stick, Patricia Agudelo-Romero, Anthony Kicic, WAERP, AREST CF

**Affiliations:** a Occupation, Environment and Safety, School of Population Health, Curtin University, Perth, Western Australia, Australia; b Wal-yan Respiratory Research Centre, Telethon Kids Institute, The University of Western Australia, Perth, Western Australia, Australia; c Department of Respiratory and Sleep Medicine, Perth Children’s Hospital, Nedlands, Western Australia, Australia; d Centre for Cell Therapy and Regenerative Medicine, School of Medicine and Pharmacology, The University of Western Australia and Harry Perkins Institute of Medical Research, Perth, Western Australia, Australia; e Australian Research Council Centre of Excellence in Plant Energy Biology, School of Molecular Sciences, The University of Western Australia, Perth, Western Australia, Australia; f European Virus Bioinformatics Center, Jena, TH, Germany; Queens College CUNY

## Abstract

Two lytic double-stranded DNA (dsDNA) bacteriophages, belonging to the family *Herelleviridae*, were isolated from wastewater in Western Australia. Biyabeda-mokiny 2 appears to belong to the genus *Kayvirus*, and Koomba-kaat 1 to *Silviavirus*.

## ANNOUNCEMENT

Antimicrobial resistance (AMR) in Staphylococcus aureus is a major threat to public health, and S. aureus is listed as a priority pathogen requiring alternative treatments by the World Health Organization (1, 2). One alternative treatment involves exploiting the lytic activity of bacterial viruses, natural bacterial predators known as bacteriophage, for direct, specific killing of S. aureus (3). Here, we report the genome sequences of two bacteriophages, Biyabeda-mokiny 2 and Koomba-kaat 1, isolated via bacterial enrichment with Staphylococcus aureus bacteria from cystic fibrosis (CF) airways.

Each bacteriophage was isolated from wastewater samples enriched with double-strength tryptic soy broth (TSB), and isolation was performed at 37°C within an orbital shaker at 50 rpm. After undergoing three rounds of plaque purification, high-titer bacteriophage lysates were then used for DNA extraction using the DNeasy Blood and Tissue kit (Qiagen) (4). Purified extractions were then sent to the Australian Genome Research Facility (AGRF) for library preparation (Nextera XT) and Illumina whole-genome sequencing using the NovaSeq 6000 platform. In total, 4,655,001 and 3,492,780 paired-end (PE) 150-bp reads were generated for Biyabeda-mokiny 2 and Koomba-kaat 1, respectively. Adaptor trimming, deduplication, and merging were performed with Trimmomatic (v0.39) (5) and BBTools (v38.96) (6). Prior to *de novo* assembly using SPAdes (v3.15.4) (7), reads were digitally normalized to a minimum of 500× and were also discarded if they had a depth of <5×. Annotations were assigned using Prokka (v1.14.6) (8) in combination with the PHROGs database (9). To calculate coverage, indexing and sorting the mapped reads were completed using SAMtools (10). Sequences were also used to check the assembly for errors using Pilon (v1.24) (11) with –drags parameter. Coverage was calculated using both the normalized and deduplicated reads using BBTools (6). Unless otherwise specified, default parameters were used for all software.

Furthermore, CheckV (v0.9.0) (12) was used to determine the presence of direct terminal repeats within both genomes and classify the genomes as “complete” with high confidence. QUAST (v5.0.2) (13) was also used to assess basic genome statistics such as percent GC content and overall length, and FastANI (v1.33) (14) was used to determine average nucleotide identity (percent ANI) (Table 1). Compared to genomes obtained using the Basic Local Alignment Search Tool (BLASTn) (15), each bacteriophage exhibited a >95% ANI score with their closest relative sequences obtained using BLASTn (Table 1). Using these data, we infer that according to published guidelines for the demarcation of taxonomic ranks (16), Biyabeda-mokiny 2 appears to belong to the genus *Kayvirus* and Koomba-kaat 1 is similar to the genus *Silviavirus*.

**TABLE 1:** Genome characteristics and comparative analysis of Biyabeda-mokiny 2 and Koomba-kaat 1 alongside their three closest relatives

Genome	Genome size (bp)	% GC	Coverage (deduplicated reads)	Coverage (normalized reads)	No. of coding sequences	Closest relative (accession no.)	Alignment (% ANI)
Biyabeda-mokiny 2	140,800	30.40	1,974×	584×	218	AP018375.1	97.0299
						KY794641.1	96.0643
						LR215719.1	96.0645

Koomba-kaat 1	135,487	30.09	1,649×	587×	184	KP881332.1	96.114
						MZ643272.1	97.1007
						KX532239.1	96.0764

All samples were collected under approval from relevant ethics committees. This article does not contain any studies involving animals performed by any author.

### Data availability.

The assembled and annotated genome is available through GenBank, under the accession numbers OP263968 and OP263969, BioProject number PRJNA862682, BioSample accession numbers SAMN30202907 (Biyabeda-mokiny 2) and SAMN30202908 (Koomba-kaat 1), and Sequence Read Archive numbers SRX17131996 (Biyabeda-mokiny 2) and SRX17131997 (Koomba-kaat 1). All scripts used for the assembly and analysis of raw reads are contained within a git repository publicly available at https://github.com/JoshuaIszatt/Phanatic.
